# A Tale of Two Nineteenth-century Dutch Jewish Hospitals—One a Success, The Other a Failure

**DOI:** 10.5041/RMMJ.10383

**Published:** 2020-10-14

**Authors:** Jack Y. Vanderhoek

**Affiliations:** Department of Biochemistry and Molecular Medicine, School of Medicine and Health Sciences, The George Washington University, Washington DC, USA

**Keywords:** Hospital, Jewish, nineteenth century, Rotterdam, The Hague, the Netherlands

## Abstract

The development of the modern hospital is usually dated to the nineteenth century. During this time, many municipal and sectarian hospitals were established and developed, and Jewish hospitals were no exception. Such developments also occurred in the Netherlands. This essay describes the different histories of the Jewish hospitals in Rotterdam and The Hague during the nineteenth century. The Rotterdam institution lasted for more than 130 years (until it was closed by the Nazis during the Second World War), whereas the one in The Hague existed for only 31 years. This study will suggest a number of possible explanations for the relatively long and successful history of the Jewish hospital in Rotterdam and the contrastingly brief duration of the Jewish hospital in The Hague.

## BACKGROUND

The nineteenth century marked the beginning of the development of the modern hospital. In the capital cities of many European countries, municipal hospitals served the needs of the poor, but there were also private, charitable institutions (or “voluntary” hospitals), usually founded by special groups, such as religious organizations, which cared for their needy ill. Jewish communities were no exception. During this time, the first Jewish hospitals were established in Austria (Vienna, 1793),^1^ England (London, 1747),^2^ France (Paris, 1836),^2^ Germany (Berlin, 1753),^2^ and the Netherlands (Amsterdam, 1804).^3^

Throughout their history in the Netherlands, most Jews (>50%) lived in Amsterdam. The cities with the next largest Jewish population numbers were Rotterdam, one of the most important Dutch ports and the second most populous city, and The Hague, the seat of the national government. A comparison of the Jewish population trends in these three cities during the nineteenth century is shown in Table 1. Hence it is not surprising that these three cities were the only ones in which Jewish hospitals were established. The Jewish hospitals in nineteenth-century Amsterdam were the subject of a previous study.^3^ However, the founding and history of the other two Jewish hospitals were quite different. The objectives of this paper are to examine nineteenth-century hospital care for Jews in Rotterdam and The Hague and to suggest possible reasons why the Jewish hospital in Rotterdam had a long and successful history for nearly a century and a half, whereas the one in The Hague only lasted for a relatively short and unimpressive 30-odd years.

**Table 1:** Selected Nineteenth-century Dutch City Jewish Population Numbers.

Years	Amsterdam4(a)	Rotterdam4(b)	The Hague4(c)	Total No.Dutch Jews^5^	Total DutchPopulation 6
1809	21,441 (54%)	2,113 (5.3%)	1,871 (4.7%)	39,596 7	2,191,000
1840	23,176	2,823	2,768	52,245	2,874,000
1859–1860 8(a)	28,389 8(a)	4,285 8(a)	4,348 8(a)	63,790	3.312,000
1869	30,039	5,297	3,651	68,003	3,560,000
1879–1880	40,318 9	6,516 10(a)	NA	81,693	4,016,000
1899	59,117 (57%)	9,019 (8.7%)	5,591 (5.4%)	103,988	5,040,000

A brief history of the Jews in these cities is essential to understanding why the Jewish hospitals in these cities developed so differently. In the early decades of the seventeenth century, Conversos from Antwerp were the first Jewish settlers in Rotterdam, and by 1647 the city fathers granted the Portuguese Jews the freedom to practice their religion, carry out wholesale and foreign trade, and to practice medicine.4(d) However, the Portuguese community lasted less than a century and ceased to exist in 1736.4(d) The second half of the seventeenth century saw the influx of Ashkenazi Jews from Eastern Europe and Germany, whose numbers grew sufficiently to establish in 1674 a congregation with a synagogue, rabbi, and cemetery.4(d) As elsewhere in Holland (and in other parts of Europe), the obstacles raised by the guilds and supported by the municipal authorities made it extremely difficult for Jews to prosper. Political changes in 1795 resulted in the legal elimination of these discriminatory economic regulations. The second half of the nineteenth century was marked by the remarkable economic growth of Rotterdam, which also led to the economic improvement of the Jewish community. The Jewish population in Rotterdam during the nineteenth century increased nearly 5-fold (from 2,000 in 1795 to 9,019 in 18994(d)), but part of this group was heavily dependent on charity for their livelihood. In 1795, more than 50% of Jews were on poor relief; in 1846, between 600 and 700 Jews (22%) were totally dependent on charity; in 1873 and 1900, the numbers were about 1,700 (31%) and about 1,600 (16%), respectively.4(e)

As Parliament and the palace of the stadtholder (national leader) were situated in The Hague, the city attracted a number of wealthy Portuguese Jewish diplomats, merchants, and physicians during the first half of the seventeenth century. However, not until the end of the century did the Jewish Portuguese community formally organize and found two synagogues. These two congregations merged about 50 years later in 1743 and lasted until the Second World War. Until the beginning of the nineteenth century, this Portuguese community was quite influential due to its wealth, as well as its national (including the House of Orange), governmental, and international connections. The rich Portuguese lived in sumptuous houses in the best neighborhood.8(b) However, terrible economic conditions in the Netherlands at the end of the eighteenth century as well as a disastrous Fourth Anglo-Dutch war (1780–1784) had a major financial impact on the Portuguese Jewish community, diminishing much of their wealth and influence. Ashkenazi Jews lived in the poor section of the city and opened their first synagogue in 1723. During the following century, their numbers surpassed those of their Portuguese co-religionists, but they tended to remain poor as they lacked the economic connections of the Portuguese community. Most were small retailers, shopkeepers, and peddlers and were barred by nearly all guilds.4(f) Only a very small percentage of the Ashkenazi community were wealthy. In 1798, the municipal authorities finally allowed the Jewish community to hold public collections for charity as other religious groups had been permitted to do for centuries.4(g) Until the 1830s, the Ashkenazi *Parnassim* were responsible for the care of their communal poor and ensured that the poor were supported in part by voluntary charitable donations and a tax on kosher meat. In 1836, the community established the Dutch Israelite (Jewish) Poor Administration of The Hague (*Nederlandsch Israelitisch Armbestuur*, NIA*)* whose responsibilities included poor aid relief, an old age home, and an orphanage.11(a) In the nineteenth century, the Jewish population in The Hague increased about 3-fold (from 1,871 to 5,591) but always remained at about 5% of the total number of Dutch Jews.

## LOCAL HOSPITALS—JEWISH AND MUNICIPAL *GASTHUIZEN*

In contrast to today, hospitals in the eighteenth and early nineteenth centuries were intended exclusively for the sick poor, as all others who were afflicted by illness would request a physician to come to their home in order to be diagnosed and treated there. Hospitals were considered a “last resort” for “patients who could no longer care for themselves and whose relatives could no longer care for them extramurally” so that “In the early 19^th^ century a Dutch hospital was not a place where one would go in order to be made well: rather it was a place to die.”^12^

During the nineteenth century, Rotterdam experienced a 6-fold increase in its population (from 53,012 in 1795 to 318,407 in 1899).^13^,^14^ During the first half of the century, the number of paupers varied between four and five thousand (5%–7% of the city population).10(b, Consequently, the need for hospital medical care, especially for its poor, also soared. Part of this need was met by the founding in 1839 of the second municipal hospital, the *Coolsingel Ziekenhuis*,15(a) but also by the establishment of hospitals associated with religious organizations and specialty hospitals during the latter half of the century.15(b) (The first municipal *Gasthuis* [almshouse] was founded in 1576 and functioned not only as a hospital where the sick poor were treated but also as a poorhouse that tended to the poor and aged.)

Poor relief was always a concern for the leadership of every Jewish community, and medical care for the indigent sick was an important aspect of the Jewish communal poor relief effort. Usually, the local Jewish community, as stated in their bylaws, would be responsible for providing (and remunerating) the services of either a physician or surgeon assigned specifically to care for the indigent patients. Though this practice continued during the nineteenth century, some Jewish patients were also treated in municipal hospitals. For example, this occurred in The Hague (see below) in 1823 and in Leeuwarden in 1865.^16^ In contrast, the Amsterdam municipal authorities refused to admit Jews to their municipal hospital in 1825.^3^

## THE JEWISH HOSPITALS IN NINETEENTH-CENTURY ROTTERDAM

At the end of the eighteenth century, the Rotterdam Jewish community had increased sufficiently so that a Jewish hospital was urgently needed, as about half of the 2,500 Jews residing in the city were paupers.17(a) This was probably because the communal leadership had concluded that the medical care for their indigent in the municipal *Gasthuis* was inadequate. As early as 1798, a committee from the Jewish community requested permission from the municipal authorities to establish a Jewish hospital, but the request was denied.17(b) However, in 1806 the Jewish community must have obtained the necessary approval, as they purchased a residence (located on the western side of the Schiedamsche Dijk) whose sole purpose was to lodge the aged poor and to house and treat the indigent Jewish sick. This *gasthuis*, the *Gesticht voor Israelitische Oude Lieden en Zieken* (Institution for the Jewish Aged and Sick) was actually the second hospital that was established in Rotterdam.15(b),17(b) It is likely that the hospital could only have been started with the approval and subsequent supervision of the all-powerful communal *Parnassim*. The hospital seems to have had a precarious financial existence, as it had to be totally supported by contributions of the synagogue members, who were mostly not wealthy. Not much is known about this institution, but in 1811 the administration of the institution included six directors, a secretary, and several matrons. However, financial problems constantly threatened its survival, and in 1820 the hospital went bankrupt. Within 7 years, the Jewish community rented another house (according to Italie, its location could not be ascertained)17(b) to serve as infirmary, but the care and nursing were so unsatisfactory that many Jewish indigents opted to use the municipal hospital. (In order to ensure that the Jewish patients were able to obtain kosher food, the *Parnassim* turned to the municipal authorities to approve the establishment within the municipal hospital of a separate facility to prepare ritually permitted food. In 1832, this request was denied, but the hospital was permitted to supply Jewish patients with kosher food prepared elsewhere.17(b))

This situation remained until 1837 when a new Jewish Hospital and Old Age Home, named *Mechon Hatsedek* (The Abode of Righteousness) was established on the Hoogstraat, wijk 10 (district 10), No. 107, at the instigation of Mr L.S. de Sterke with the support of the *Parnassim* and Chief Rabbi E.J. Lowenstam.18(a) The cost of acquiring the new hospital was covered by the *Israelitische gemeente* (Jewish congregation) as well as private donations from both Jewish and non-Jewish residents.18(b) The new institution was supervised by the *Parnassim*, with the medical care managed by Dr L. Levie, physician, and Dr P.J. van Wageningen, surgeon. However, the situation changed in January 1838.

In the early decades of the nineteenth century, the national government mandated that the Dutch Jewish community be reorganized whereby the *Parnassim* lost most of their power outside the synagogue, including supervision of all Jewish poor relief activities. The management of these relief activities, including medical care for indigent Jews, was assumed by the newly established (1838) Rotterdam Dutch Israelite (Jewish) Poor Administration (*Nederlandsch Israelitisch Armbestuur*, NIA). As a result, Messrs Ezechiels, Levisson, and van Raalte, governors of the NIA, were assigned in 1838 to manage the *Mechon Hatsedek*.18(b) From 1838 to 1840, financial and domestic matters, institutional regulations, salaries, etc. were regulated.^19^ In 1840, a newly passed law required that poor Jewish elderly were also to be housed in hospitals, and so the *Mechon Hatsedek* became the Israelite (Jewish) Home for the Elderly and Sick. (It should be noted that although some needy elderly were always housed in the institution before 1840, the new law made the sheltering of the indigent aged in this institution compulsory.)

**Figure 1:**
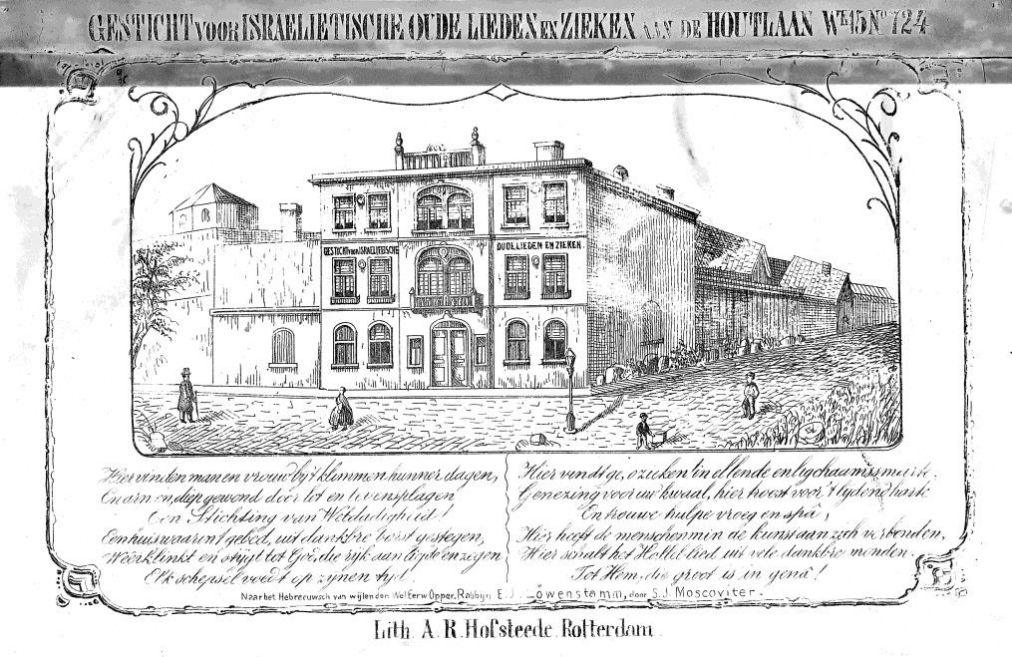
View of *Mechon Hatsedek*, the Rotterdam Jewish Home for the Aged and Sick, on the Houtlaan No. 728, in 1858–1862.^21^

An increase of the number of Jews in Rotterdam (from 3,300 in 1837 to 4,800 in 1865) was accompanied by a substantial rise in the number of destitute Jews (from 6–700 in 1848 to 1,600–1,700 between 1873 and 1900).^20^ Consequently, *Mechon Hatsedek* had to be expanded, and in 1864 a suitable opportunity presented itself whereby a house with suitable adjacent plots of land located on the Houtlaan (wijk 14), No. 728 was acquired.^20^ The financial support of the Jewish community as well as non-Jewish benefactors was again crucial, as *Mechon Hatsedek* itself did not have sufficient financial resources. The building opened its doors on April 15, 1866, and a picture of this institution is shown in Figure 1.

Despite the fact that part of *Mechon Hatsedek* was intended to care for the indigent Jewish sick,^19^,^22^ the institution could not satisfy its goals, as it neither could offer medical treatment to those who were not associated with the establishment (e.g. patients with venereal or hereditary diseases were not admitted)^22^ nor could it provide a suitable place to carry out medical operations. Surgeries as well as the treatment of seriously ill patients had to be carried out in one of the municipal hospitals. Finally, in 1893, at the instigation of the house surgeon, M. Polak, as well as the support of the Regents of the Home, an operating room was finally equipped, more patients were accepted, and a licensed nurse replaced an unschooled aide. Table 2 compares the number of patients admitted to the *Mechon Hatsedek* hospital during three key periods of its history: from 1840 to 1856 at the Hoogstraat location, from 1890 to 1898 at the Houtlaan site and from 1901 to 1914 at the Schietbaanlaan.15(c),18(c) However, it was clear that the home just did not have sufficient space for all its essential endeavors. After receiving a number of substantial donations, it was decided in 1899 to purchase a new parcel of land that would allow the establishment of the *Mechon Hatsedek* hospital, with an entrance on the Schietbaanlaan No. 42 and a separate Jewish Old Age home located around the corner on the Claes de Vrieslaan. These institutions were inaugurated on October 22, 1900. The hospital contained a large as well as a small operating room, four large wards, and rooms for private patients.17(c) Throughout its existence, it had to surmount serious financial problems. However, the hospital managed to incorporate the latest medical advances and treated all patients except those with contagious diseases or the mentally ill. The medical staff consisted of the physician J. Voorzanger, the surgeon M. Polak, a head nurse, and six nurses. A substantial increase in admitted patients was observed in this facility in the first 15 years of the twentieth century (Table 2). However, the hospital ceased to function in 1943 when the German occupiers deported both patients and staff to be murdered in various Nazi concentration camps.

**Table 2:** Patient Numbers at the *Mechon Hatsedek* Hospital in Rotterdam.

Location	Years	Patients
**At the Hoogstraat**18(c)	1840–1842	80
	1842–1845	112
	1845–1848	80
	1848–1850	116
	1851–1853	106
	1854–1856	126
**At the Houtlaan**15(c)	1890–1892	195
	1893–1895	248
	1896–1898	184
**At the Schietbaanlaan**17(c)	1901	143
	1905	228
	1909	326 23
	1914	406 24

## HOSPITAL CARE FOR NINETEENTH-CENTURY JEWS IN THE HAGUE

The only report of a *gasthuis* for the indigent ill with non-contagious diseases in The Hague dates from 1355, and it is unknown how long it survived.25(a) Until the nineteenth century the only hospitals in The Hague seem to have been several “specialty” *gasthuizen* which either focused on the prevention of communicable diseases (pest- and leper-houses) or the treatment or housing of special groups such as soldiers or the mentally ill. Not until 1815 was a house on the Koediefstraat purchased to treat the sick city poor. However, it soon became apparent that this building was much too small to meet the requirements of the local needy. A much larger residence, located on the Zuidwal, with a substantial garden, was bought in 1821 and opened its doors two years later as the municipal *Gasthuis*. The first floor was for male patients and included an operating room and wards for surgical patients and those suffering from venereal diseases or scabies, whereas the second floor was designed for female patients.25(b)

An unusual aspect of The Hague municipal *Gasthuis* was that Jewish patients were admitted right after its opening. As early as October 1823, special permission was obtained from the municipal magistrates so that a Jewish patient was allowed to receive ritually prepared food (prepared elsewhere).25(c) Although no Jewish patients seem to have been admitted from 1824 to 1828,^26^ the Jewish communal leadership decided to negotiate special arrangements for Jewish patients with the municipal authorities and the Regents of this *Gasthuis*. Thus, it was agreed to let the *Parnassim* provide ritually prepared food to the Jewish patients, to allow Jewish representatives to be present during operations, and to permit the removal of Jewish corpses for burial in the Jewish cemetery. All these changes were codified as six added Articles to the official Regulations of the Municipal *Gasthuis*.^27^ In 1829, separate wards were provided for male and female Jewish patients, as well as a Jewish cook to prepare the food.25(c) However, the number of Jewish patients admitted in the period from 1829 to 1849 showed a steady decrease as follows: starting from 40 in 1829, the number decreased to an annual average of 20 during 1832–1837 and a further decrease to an average of 14 per year during 1842–1849.^28^ It seems that the availability of kosher food was not enough of an attraction to the *Gasthuis* (apparently home medical care was preferred) despite the fact that during this period (from 1830 to 1850) the number of Jews in The Hague increased from 2,268 to 3,319. In 1861, these separate Jewish wards were abolished as their space was needed for the increasing numbers of admitted (non-Jewish) patients. In the early 1860s, the hospital was treating between 700 and 900 patients.25(d) It was quite clear to the Regents that this building had become too small to satisfy the increasing need for additional wards for patients with contagious, eye, and syphilitic diseases, convalescents, and more operating rooms. Consequently, a new and larger building was opened on the same site in 1865.25(e)

**Figure 2:**
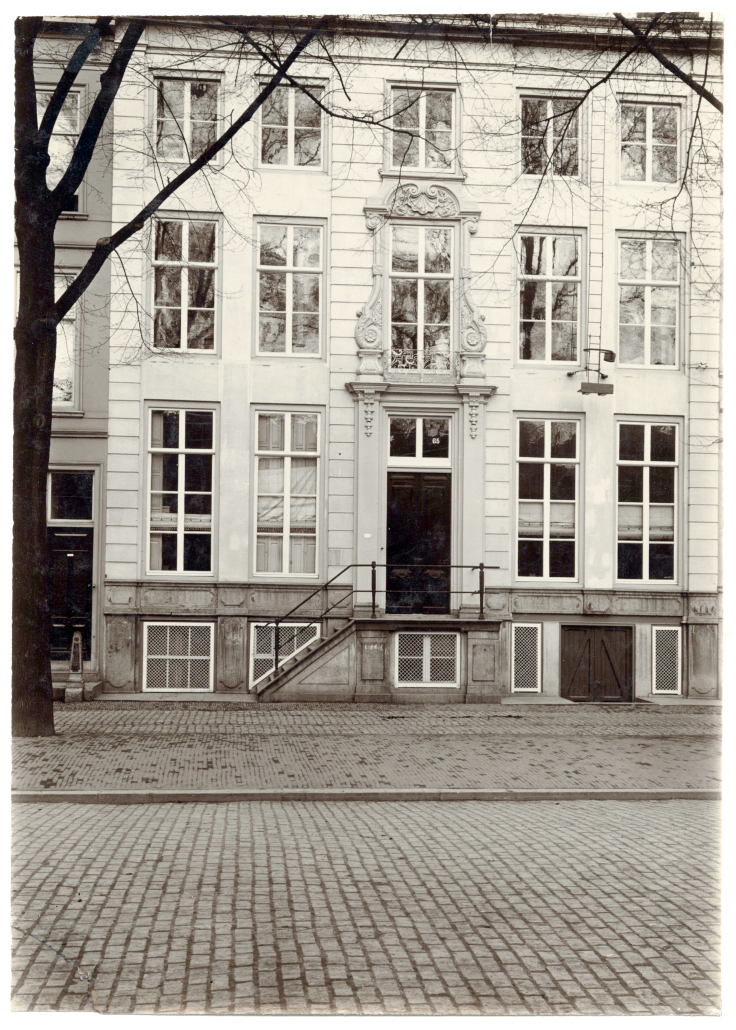
Photograph of the Jewish Hospital on the Prinsegracht 65 in The Hague in the Early 1900s.^32^

The lack of separate Jewish wards seems to have been a major impetus to consider establishing a Jewish hospital in The Hague, since many indigent ill Jews reportedly preferred to be poorly cared for, even in an attic room, than to go to a municipal hospital which would not arrange for the appropriate religious ceremonies in case (s)he died.29(a) However, it would take 10 years before such a hospital could be established. On January 15, 1871, a group of 11 individuals, including Chief Rabbis B.S. Berenstein and J. van J. Ferares, formed an official committee to establish an institution to care for the sick of both the Ashkenazi (*Hoogduitse*) and Sefardi (Portuguese) communities. However, opinions in the Jewish community regarding the need for such a hospital were quite divided. The opponents argued that the municipal *Gasthuis* was willing to accommodate Jewish patients (the municipal authorities had so declared) and that a Jewish hospital would represent the idea of Jewish separateness, isolation, and intolerance.29(b) Even the synagogue administrators, who took a strictly neutral position, did not at all cooperate and only allowed individuals to offer charity in the synagogue for the benefit of the hospital.^30^ Despite this controversy, a house with large, airy rooms and a beautiful garden, located at Prinsegracht 65, was bought in September 1871.29(c) However, at this point, there was not enough money to furnish the building with equipment needed for a hospital. Additional requests for financial support, including that from the NIA, were successful, and, coupled with an unexpected large legacy of *f* 30,000,31(a) finally enough funds were raised so that the Jewish hospital could open its doors for patients on August 10, 1873. A picture of this building is shown in Figure 2. The hospital was run by a Board of nine Regents and four Lady Regents, as well as the Chief Rabbis of the Ashkenazi and Sefardi congregations. Interestingly, the municipal authorities were not very supportive. For example, the municipal poor relief organization refused to provide either medical assistance or free medicines for patients in the Jewish hospital, even though this was their responsibility (only in 1895 did the municipal authorities agree to provide free medicines to the hospital patients). Table 3 shows the number of admitted patients over a 15-year period (from 1883 to 1897) with an annual average of 72 admitted patients during the decade from 1883 to 1892. However, by 1894, the number of admitted patients started to drop drastically. This decline, coupled with a dearth of funds needed to implement the increasing technological and personnel demands required for a hospital in the early twentieth century, led to the decision to close the hospital in 1904.25(f) At this time, the hospital Board made an agreement with the municipal authorities whereby Jewish patients would be treated in the municipal *Gasthuis* in wards designated exclusively for Jewish patients, where they would always have a Jewish nurse available and where the patients would receive ritually prepared food that would be supervised by the Rabbinical authorities.25(f)

**Table 3:** Patient Numbers at the Israelitisch Ziekenhuis (hospital) in The Hague.31(b)

Years	Patients
1883	63
1884	79
1885	58
1886	64
1887	57
1888	90
1889	75
1890	89
1891	71
1892	73
1894	45 (average)
1895	34
1897	40 33
1904	0 (closed)

## POSSIBLE REASONS FOR JEWISH HOSPITAL LONGEVITY SUCCESS IN ROTTERDAM AND FAILURE IN THE HAGUE

In view of the above discussion, there are several possible rationalizations for the different developments of the Jewish hospitals in Rotterdam and The Hague.

During the eighteenth and first half of the nineteenth century, most of the Rotterdammers were poor, and the situation was even worse for the Jewish inhabitants of the city since they were banned from most trades. Hence it is not surprising that this situation, coupled with the typical insularity of most Jewish communities of the time, resulted in a community that was totally dependent on its own resources. Since caring for the sick Jewish indigent was an important religious obligation, treating them in a Jewish hospital had many advantages such as providing the patients with kosher food and religious ceremonies and being among their own people during these hard times. It seems that as early as 1798 several community members felt that there was an urgent need for a Jewish hospital. This suggests that at the end of the eighteenth century the existing municipal *Gasthuis* did not adequately meet the needs, especially the religious requirements, of the destitute Jewish ill. Furthermore, the idea of a Jewish hospital in Rotterdam seemed to have preceded similar considerations made in 1802 in the Amsterdam Jewish community. From this early concern onwards, there seemed to always have been a core group of members within the Rotterdam Jewish community who created and maintained a strong tradition to ensure that their city always had a Jewish hospital. Overt opposition was not evident. As a result, very few Jews opted to use the municipal hospitals during the nineteenth century.15(d) Except for a seven-year hiatus (from 1820 to 1827), this Jewish community, either directly, through the NIA, or with some municipal support (especially in the twentieth century), managed to provide sufficient financial support to care for their needy ill in a Jewish hospital for nearly a century and a half (1806–1943).

The situation in The Hague was different. It seems that the Jews in The Hague were probably more influential than their co-religionists in Rotterdam. This may have contributed to the fact that the relations between the municipal authorities and the Jewish communal leadership were quite satisfactory. For example, when the Regents of the Surgeon’s Guild objected to allowing a Jew to become an apprentice to a Guild member, the Mayor and councilors overruled these objections at the urging of the community’s *Parnassim*.^34^ In addition, a number of Jewish physicians and surgeons in The Hague (and elsewhere in Holland) had both Jewish as well as a number of prominent Christian patients.^35^ Finally, the influence of the ruling House of Orange cannot be discounted, since its rulers, who were located in The Hague for most of the eighteenth and nineteenth centuries, were very sympathetic to the Dutch Jewish community, including those in The Hague. All these elements may have led the municipal magistrates to grant special permission for a Jewish patient to receive kosher food in the new municipal *Gasthuis* shortly after it opened in 1823.25(c) Several years later, the Jewish communal leadership negotiated a more extensive agreement including separate wards for Jewish patients (see above). In view of this arrangement, there seemed to have been less impetus for the establishment of a Jewish hospital. However, when separate Jewish wards in the municipal *Gasthuis* were abolished (see above), this undoubtedly changed the relations between the hospital and the Jewish community. This in turn impelled a group of individuals, including both Chief Rabbis, to try and establish a Jewish hospital in The Hague. However, there was strong opposition within the Jewish community to this suggestion. These opponents argued that such an institution was unnecessary since the municipal *Gasthuis* would be willing and able to meet the religious needs of the Jewish patients. Even though the Jewish hospital opened in 1873, its financial support was probably weaker than it could have been due to this intra-communal dispute; this ultimately led to the closure of The Hague Jewish hospital after only 31 years of existence.

Why was there such strong support within the Jewish community for using the municipal hospital facilities for Jewish patients? First of all, the financial angle cannot be dismissed, as it was probably much less expensive to provide support for Jewish patients in an established municipal institution than to maintain an entire Jewish hospital, its staff, instruments, upkeep, etc. When it became essential to upgrade its aged facilities around the beginning of the twentieth century to meet the increasing technical demands for an up-to-date hospital, the lack of sufficient funds and receptive support doomed this institution. Second, to maintain an “all-Jewish” environment (in a Jewish hospital) seemed less important to a substantial part of the community (in apparent contrast to their charitable co-religionist benefactors in Rotterdam) so that they did not provide monetary support for this charitable institution. Third, it seems that the friendly attitude of both the municipal and *Gasthuis* authorities was also very important in attracting Jewish patients to this institution. Finally, the refusal of the municipal poor administration to provide medical care and free medicines to patients in the Jewish hospital also played into the hands of the opponents.^30^

Another group possibly opposed to establishing a Jewish hospital might have been the *Bikur Cholim* (visiting the sick) society in The Hague. The primary function of a communal *Bikur Cholim* charitable association was to visit the sick (usually in their homes) and, if necessary, to provide help in obtaining medical care. *Bikur Cholim* societies have been very common in nearly all Jewish communities throughout the ages.^36^ The main revenue for the *Bikur Cholim* charities came from donations and legacies. In Rotterdam, a *Bikur Cholim* society that helped in the care of the needy ill in their homes had been reported during the eighteenth century, but very little is known about it. However, this group had ceased to function by the middle of the nineteenth century as it no longer was included in the list of Jewish charitable organizations in Rotterdam.17(b),18(d)

There is already a mention of a *Bikur Cholim* association in The Hague during the eighteenth century,^28^ and it seems that the charity remained active in the nineteenth century.11(b,c),^37^ In 1828, the governing Board of The Hague Jewish community decided to make this charity responsible for the care of the Jewish patients, including its indigent (e.g. provide kosher food and free medicines), either in their homes or in the municipal *Gasthuis*.11(c) It would not have been very hard to predict that the establishment of a Jewish hospital would have a negative impact on the many responsibilities of the *Bikur Cholim*, and this would result in a greatly diminished influence of the charity within the Jewish community. It would not have been surprising if the leaders of this society may have tried to undermine the hospital project. This might explain why the communal leaders (perhaps to circumvent this presumed “opposition”) decided to grant the *Bikur Cholim* organization two representatives on the governing Board of the Jewish hospital.31(c) (In return, the *Bikur Cholim* society also agreed to pay the hospital one-third of the annual patient care.11(c))

The closure of the hospital in 1904 led to increased responsibilities for the *Bikur Cholim* society, especially in the municipal *Gasthuis*, and the restoration of its influence certainly would have been a very welcome development.

It is noteworthy that the founding of the Jewish hospital in The Hague (though not in Rotterdam) coincided with the rapid rise in religious-based hospitals in the second half of the nineteenth century in Holland. For example, six such institutions were established in Rotterdam and two in The Hague.15(b) Perhaps one goal for these different religious communities was to maintain a religious cohesiveness within their group. This approach was obviously not very successful in the case of the short-lived Jewish hospital in The Hague.

This history of the Jewish hospitals in Rotterdam and The Hague is a nineteenth-century cautionary tale, especially in terms of longevity. The Rotterdam Jewish hospital, without major controversy, lasted for nearly a century and a half, but dissensions within The Hague Jewish community were a major reason for the barely 30-year existence of its hospital.
